# An Occult Odontogenic Infection Behind a Persistent Left Chin Nodule

**DOI:** 10.1002/kjm2.70243

**Published:** 2026-06-10

**Authors:** Yi‐Shan Teng, Huei‐Jing Wang, Shih‐Tsung Cheng

**Affiliations:** ^1^ Department of Dermatology Kaohsiung Medical University Hospital, Kaohsiung Medical University Kaohsiung Taiwan; ^2^ Department of Dermatology, College of Medicine Kaohsiung Medical University Kaohsiung Taiwan

1

A 66‐year‐old woman was referred to our dermatology clinic for a painful lesion on her left lower chin that had persisted for 3 months. She had received several courses of oral antibiotics without improvement. She recalled transient dental pain 4 months earlier and had undergone root canal treatment. Examination revealed a tender, protruding erythematous nodule with peripheral dimpling on the left lower chin (Figure 1A) and a corresponding ulceration on the adjacent gingival mucosa (Figure 1B). The lesion intermittently drained seropurulent material. Skin biopsy showed abscess formation with granulomatous inflammation but did not explain the chronic course. Given the persistent lesion and dental history, a panoramic radiograph was obtained and demonstrated a periapical radiolucency at the left mandibular canine—the tooth treated 4 months earlier (Figure 1C). A diagnosis of chronic periapical infection with formation of an odontogenic cutaneous fistula (OCF) was made. The patient subsequently underwent extraction of the affected tooth with fistulectomy, resulting in complete resolution within 1 month and no recurrence at three‐month follow‐up.

**FIGURE 1:**
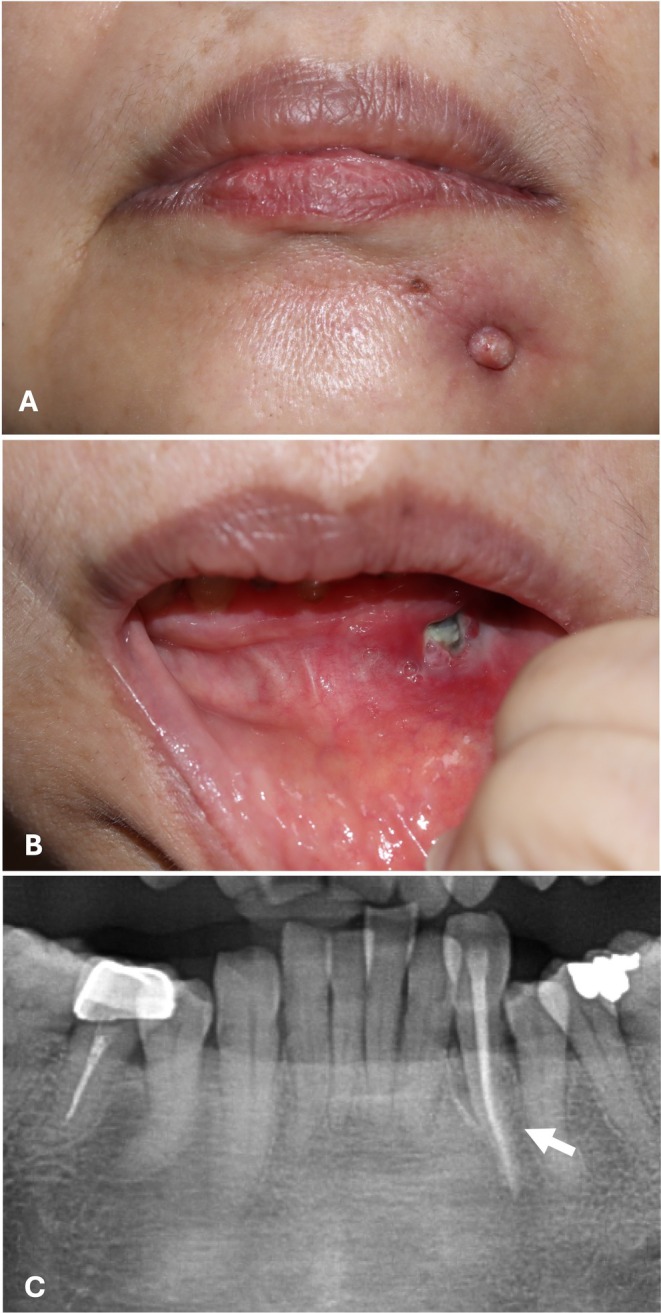
(A) an erythematous, protruding nodule with peripheral dimpling on the left lower chin. (B) A corresponding ulceration on the gingival mucosa. (C) A radiograph demonstrating an endodontic treatment with a radiolucent lesion in the periapical area at the site of the affected tooth.

OCFs are uncommon but clinically significant and frequently misdiagnosed [1]. Chronic dental infection is the most common cause, although trauma, implants, osteomyelitis, medication‐related osteonecrosis, and malignancy may also be responsible. Because many patients lack dental pain—especially in chronic infections with pulpal necrosis—OCFs frequently mimic primary dermatologic conditions. Their cutaneous manifestations vary widely, including dimpling, nodules, abscesses, cysts, ulcers, draining tracts, or nodulocystic lesions. This wide morphological spectrum explains why OCF are commonly mistaken for epidermal cysts, furuncles, basal cell carcinoma, squamous cell carcinoma, or infectious granulomas [2, 3]. Consequently, patients often undergo unnecessary biopsies, repeated antibiotics, or destructive procedures before the dental origin is recognized. Recognized risk factors include poor oral hygiene, failed or incomplete endodontic therapy, xerostomia, and immunocompromised states [1]. Diagnosis requires a high index of suspicion supported by dental history, careful intraoral examination, and imaging. Subtle mucosal openings may be the only clue to periapical disease, while radiography or computed tomography helps identify the tract and underlying pathology. Gutta‐percha tracing through the cutaneous opening can help confirm the communication [1, 4, 5]. Definitive treatment involves addressing the source tooth through extraction or endodontic retreatment [5]. Cutaneous lesions usually resolve with minimal scarring once the dental infection is eradicated. Early recognition is therefore essential to prevent unnecessary procedures, reduce morbidity, and improve patient outcomes.

This case highlights the importance of maintaining a high index of suspicion for odontogenic sources when evaluating persistent or atypical lesions of the lower face. Systematic assessment—including detailed dental history, meticulous intraoral examination, and appropriate radiographic imaging—remains essential for timely and accurate diagnosis. Strengthening collaboration between dermatology and dental disciplines not only facilitates comprehensive evaluation but also ensures that underlying pathology is addressed at its source. Incorporating these principles into clinical practice may reduce diagnostic delays, prevent unnecessary interventions, and ultimately improve patient outcomes in cases of odontogenic cutaneous fistulas.

## Conflicts of Interest

The authors declare no conflicts of interest.

## Data Availability

The data that support the findings of this study are available from the corresponding author upon reasonable request.
